# Atomistic Engineering of Phonons in Functional Oxide Heterostructures

**DOI:** 10.1002/advs.202103403

**Published:** 2022-01-17

**Authors:** Seung Gyo Jeong, Ambrose Seo, Woo Seok Choi

**Affiliations:** ^1^ Department of Physics Sungkyunkwan University Suwon 16419 Korea; ^2^ Department of Physics and Astronomy University of Kentucky Lexington KY 40506 USA

**Keywords:** atomic‐scale epitaxy, confocal Raman spectroscopy, engineering of phonon, synthetic oxide crystal, terahertz acoustic wave

## Abstract

Engineering of phonons, that is, collective lattice vibrations in crystals, is essential for manipulating physical properties of materials such as thermal transport, electron‐phonon interaction, confinement of lattice vibration, and optical polarization. Most approaches to phonon‐engineering have been largely limited to the high‐quality heterostructures of III–V compound semiconductors. Yet, artificial engineering of phonons in a variety of materials with functional properties, such as complex oxides, will yield unprecedented applications of coherent tunable phonons in future quantum acoustic devices. In this study, artificial engineering of phonons in the atomic‐scale SrRuO_3_/SrTiO_3_ superlattices is demonstrated, wherein tunable phonon modes are observed via confocal Raman spectroscopy. In particular, the coherent superlattices led to the backfolding of acoustic phonon dispersion, resulting in zone‐folded acoustic phonons in the THz frequency domain. The frequencies can be largely tuned from 1 to 2 THz via atomic‐scale precision thickness control. In addition, a polar optical phonon originating from the local inversion symmetry breaking in the artificial oxide superlattices is observed, exhibiting emergent functionality. The approach of atomic‐scale heterostructuring of complex oxides will vastly expand material systems for quantum acoustic devices, especially with the viability of functionality integration.

## Graphical abstract

Atomistic engineering of functional phonons is achieved via atomic‐scale precision epitaxy in complex perovskite oxide superlattices. Zone‐folded acoustic phonons in the THz frequency domain can be created and modulated. In addition, polar optical phonons are stabilized manifesting inversion symmetry breaking in the superlattices. Tunable and coherent functional phonons realized in this study will serve as important components for future quantum acoustic applications.

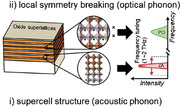

## 1 Introduction

Artificial engineering of quantized lattice vibrations, that is, phonons, is a fascinating subject in both fundamental research and practical applications.^[^ ^1^, ^2^, ^3^, ^4^, ^5^, ^6^, ^7^, ^8^, ^9^, ^10^, ^11^, ^12^ ^]^ It involves the creation and manipulation of new phonon modes through the synthesis of artificial crystals such as nanostructures of heterostructures with dissimilar materials. By manipulating the phonon dispersion, the group velocity, electric polarization, and density of states of the phonons could be effectively controlled.^[^ ^1^ ^]^ Modulations of low‐dimensional artificial nanostructures and crystals provide unique opportunities for controlling phonon and its related applications.^[^ ^2^, ^3^, ^4^, ^5^ ^]^ In particular, these approaches will pave the way for quantum acoustic applications such as quantum Bragg mirrors and cavities,^[^ ^6^, ^7^, ^8^ ^]^ quantum acoustic memory and transducers,^[^ ^9^ ^]^ microwave‐optical converters,^[^ ^10^ ^]^ quantum amplifiers,^[^ ^11^ ^]^ and circuit quantum acoustodynamics.^[^ ^6^ ^]^ The quantized ground state phonon is envisaged as a potential resonator for managing quantum information, for example, superconducting quantum bits,^[^ ^9^ ^]^ implying that facile engineering of the low‐frequency phonons is highly necessary. However, most studies on artificial phonon engineering have been exclusively focused on compound semiconductor heterostructures,^[^ ^7^, ^8^, ^13^, ^14^ ^]^ partially owing to their excellent crystalline qualities.

Complex perovskite oxide thin films and heterostructures provide accessible controllability of functional phenomena, which are closely associated with phonon dynamics.^[^ ^15^, ^16^, ^17^, ^18^, ^19^, ^20^, ^21^, ^22^, ^23^, ^24^, ^25^ ^]^ Modulation of lattice structures by epitaxial strain causes a substantial shift in phonon wavelength.^[^ ^17^, ^18^ ^]^ Adjustable soft‐mode phonons can further lead to an enhanced dielectric constant and emergence of ferroelectricity in perovskite oxides.^[^ ^19^, ^20^ ^]^ Superlattices composed of perovskite oxides have also been shown to systematically reduce the thermal conductivity or enhance electron‐phonon coupling for thermoelectric purposes.^[^ ^21^, ^22^ ^]^ Last but not least, phonon excitation energy in perovskite oxides has similar values with various quasiparticles including plasmons, excitons, phasons, magnons, and skyrmions, representing a possible correlation to the phonon dynamics.^[^ ^25^ ^]^

**Figure 1:**
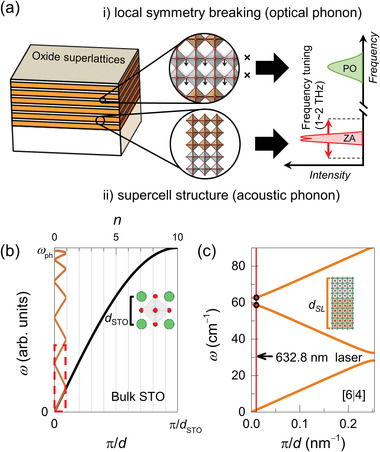
Phonon engineering in artificial oxide superlattices. a) Schematic representation of the emergent phonons in atomically designed oxide superlattices. i) Artificial superlattice structure realizes local symmetry breaking at the interfacial layers, denoted by the crosses. As a result, new PO phonon modes can be stabilized and observed. ii) The supercell structure leads to backfolding of the phonon dispersion, activating a tunable THz excitation of the ZA phonon modes. b) Schematic representation of the evolution of ZA phonon modes in SRO/STO superlattices when *d*_SL_ = 10 *d*_STO_. The inset shows the atomic u.c. of STO with *d*_STO_. c) Estimated acoustic phonon dispersion of [6|4] superlattice with *d*_SL_ at low photon energy region (red dotted box region in (b)). The vertical red line shows the *q* value of probing laser with a wavelength of 632.8 nm.

Both optical and acoustic phonons can promote intriguing phonon‐related phenomena in perovskite oxide heterostructures, as shown in **Figure** **1**a. i) First, artificial superlattice structures can break the local inversion symmetry.^[^ ^24^, ^26^, ^27^ ^]^ For example, the inversion symmetry is broken from the perspective of the interfacial gray octahedral layers shown in Figure 1a, which might induce a polar optical (PO) phonon.^[^ ^28^ ^]^ Because of the absence of inversion symmetry, such phonons are intrinsically polar. ii) Second, acoustic phonon dispersion can be controlled by the supercell periodicity of the superlattice. The supercell structure induces backfolding of the phonon dispersion from that of the bulk, leading to a reduced Brillouin zone and tunable zone‐folded acoustic (ZA) phonons.

Figure 1b schematically shows the phonon dispersion of a bulk material (SrTiO_3_, STO) and a superlattice (SrRuO_3_/SrTiO_3_, SRO/STO superlattice). The phonon frequency of STO is given by, *ω* ≅ sin(*kd*_STO_/2), where *d*_STO_ and *k* = *π*/*d* are the lattice constants of the bulk STO in the real and reciprocal spaces, respectively (black curve in Figure 1b). In contrast, the value of *k* for a superlattice with a supercell periodicity (*d*_SL_) of, for example, 10 *d*_STO_, decreases by 10 times, and equivalent backfolding of the Brillouin zone appears (orange curves in Figure 1b). This leads to Raman‐active zone‐center acoustic phonons near the Γ‐point (Figure 1c). The ZA phonon dispersion of the superlattice with the reduced Brillouin zone, shown in Figure 1c, is estimated using Rytov approximation as^[^ ^13^ ^]^

(1) $$\begin{array}{c} \begin{array}{c} \cos\left(kd_{\mathrm{SL}}\right)=\cos\left(\frac{\omega d_{1}}{v_{s1}}\right)\cos\left(\;\frac{\omega d_{2}}{v_{s2}}\;\right)\;-\;\frac{1+\alpha^{2}}{2\alpha }\sin\left(\;\frac{\omega d_{1}}{v_{s1}}\;\right)\sin\left(\frac{\omega d_{2}}{v_{s2}}\right) \end{array} \end{array}$$

where *d*_1_ and *d*_2_ are the thicknesses and *υ*_s1_ and *υ*_s2_ are the sound velocities of the two distinct layers within the superlattices. The parameter *α* is defined as *α* = *υ*_s2_ *ρ*_2_ / *υ*_s1_ *ρ*_1,_ where *ρ*_1_ and *ρ*_2_ are the densities of the two materials, respectively. If each atomically thin layer within the superlattices well‐preserve its sound velocity and density, the Rytov model would be also valid for atomically thin heterostructures. As an example, we modeled the six‐ and four‐unit cell (u.c. ∼0.4 nm) layers of the SRO and STO layers within a superlattice, that is, [6|4] superlattice (*d*_SL_ = 10*d*_STO_), and used the reported *υ* and *ρ* values of the individual SRO and STO materials.^[^ ^29^, ^30^, ^31^, ^32^ ^]^ The phonon dispersion of the superlattice exhibits the measurable peak frequencies (*ω*_SL_) of ZA phonons at ∼60 cm^−1^ (∼1.8 THz) with a laser wavelength of 632.8 nm for Raman spectroscopy. Furthermore, it shows a tunable *ω*_SL_ via the atomically designed supercell thickness (*d*_1_ + *d*_2_) of the superlattices.

In this study, we report the atomic‐scale precision modulation of phonon behavior (both optical and acoustic) using artificial oxide superlattices. We chose the SRO/STO superlattices as a model system to realize artificial phonon engineering. STO is an incipient ferroelectric, where the PO phonons are closely associated with its quantum paraelectric phenomena.^[^ ^33^, ^34^ ^]^ The atomically sharp interfaces and surfaces of the SRO/STO superlattices led to the facile engineering of the phonons.^[^ ^35^, ^36^, ^37^, ^38^, ^39^, ^40^ ^]^ The superlattices successfully realized THz excitations of ZA phonon modes and largely modulated the excitation frequency up to 2 THz via atomic‐scale epitaxy. Furthermore, the superlattice structure exhibited a PO phonon mode, which was not observed in either bulk STO or SRO, indicating local inversion symmetry breaking. Our result demonstrates a novel route for the artificial engineering of functional phonons in complex oxide heterostructures, which would be useful for designing optical transducers in the THz region, in conjunction with the piezoelectricity and deformation potential effects.^[^ ^41^, ^42^ ^]^ Furthermore, the THz frequency domain has tremendous potential for the development of next‐generation communication devices due to its higher data transfer rate.^[^ ^43^, ^44^ ^]^

## 2 Results and Discussion

### 2.1 High‐Quality Epitaxial Oxide Superlattices

**Figure 2:**
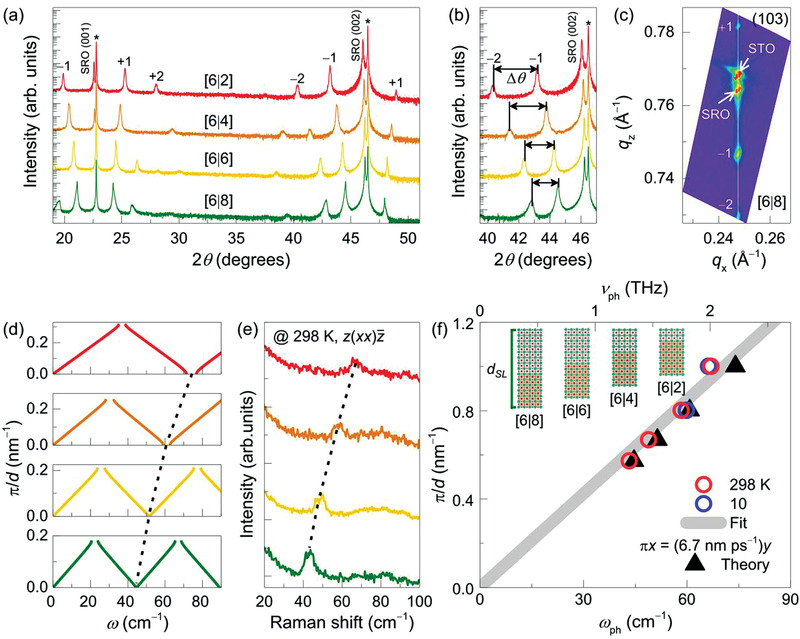
Atomic‐scale phonon modulation in SRO/STO superlattices. a,b) XRD *θ*–2*θ* measurements are shown for [6|*y*] with different *y*, grown on (001)‐oriented STO substrates. The asterisks (*) indicate the (00*l*) Bragg peaks of the STO substrates. The Bragg peaks of the superlattice (±*n*) with ∆*θ* represent well‐defined periodicity. c) XRD RSM is shown for [6|8] superlattice, around the (103) Bragg reflections of the STO substrate. d) Simulated phonon dispersions and e) Raman spectra are shown for ZA phonon modes of superlattices with different *d*_SL_. The spectra are measured with *z*(*xx*)$\bar{z}$ scattering configuration at room temperature. The dotted lines are shown as a guide to the eye. f) Phonon dispersion for the low‐energy region is estimated using *ω*_ph_ of [6|*y*] with different *y*. The solid line represents the fit of *ω*_ph_ at room temperature.

High‐quality epitaxial SRO/STO superlattices with systematically modulated supercell thicknesses were synthesized using pulsed laser epitaxy (**Figure** **2**a–2c and Figures S1 and S2, Supporting Information). We fabricated the six‐ and *y*‐u.c. of SRO and STO layers with 50 repetitions on (001) STO substrates, that is, [6|*y*] superlattices. The number of u.c. of the superlattices was controlled utilizing a customized automatic laser pulse control system.^[^ ^35^, ^36^, ^45^, ^46^, ^47^ ^]^ X‐ray diffraction (XRD) *θ*–2*θ* scans showed clear superlattice peaks (±*n*) with Pendellösung fringes, indicating atomically well‐defined supercell structures (Figure 2a,b). The XRD reciprocal space maps, as shown in Figure 2c and Figure S1, Supporting Information, exhibited fully strained superlattices. Previous scanning tunneling electron microscopy has also shown the atomically sharp interfaces of our SRO/STO superlattices.^[^ ^35^, ^36^ ^]^ The coherent supercell structures, even at 50 repetitions (thicknesses of 160–280 nm), enhance the inelastic light scattering cross‐section sufficient for the experimental measurement and practical utilization of consistent phonon modes.

### 2.2 Tunable Zone‐Folded Acoustic Phonon Modes

Precise thickness control manifests zone‐folding of acoustic phonon modes with fine‐tuned THz frequencies. Figure 2b shows that the angular separation between the neighboring superlattice peaks (Δ*θ*) systematically decreases with increasing *y*, indicating an increased *d*_SL_. The *d*_SL_ was characterized by Bragg's law as

(2) $$d_{\mathrm{SL}}\;=\;\frac{\lambda }{2}{\left(\sin\theta_{n}-\sin\theta_{n-1}\right)}^{-1}$$

where *λ*, *n*, and *θ_n_* are the wavelength of the X‐ray (0.154 nm for Cu K*‐α*_1_), the order of superlattice peaks, and the *n*th‐order superlattice peak position, respectively. The estimated *d*_SL_ values for [6|*y*] superlattices with *y* = 2, 4, 6, and 8 are 3.16, 3.80, 4.70, and 5.48 nm, respectively, closely matching the target thickness with a deviation smaller than 0.20 nm. Figure 2d shows the acoustic phonon dispersion of SRO/STO superlattices in the mini‐Brillouin zone, modeled by Rytov approximation. With increasing *y* from 2 to 8, the estimated *ω*_ph_ of ZA phonons is systematically reduced from 74 to 45 cm^−1^ (2.2 to 1.3 THz). This suggests that the *d*_SL_ of the atomically well‐defined superlattice is an effective control parameter for modulating the *ω*_ph_ of ZA phonons.

The theoretically estimated *ω*_ph_ (Figure 2d) was directly observed via confocal Raman spectroscopy, as shown in Figure 2e and Figure S3, Supporting Information. Clear Raman excitations of ZA phonons were found with the modulation of their *ω*_ph_ systematically depending on *y*. Both Stokes and anti‐Stokes shifts of the zone‐folded phonons were observed for [6|8] superlattice, as shown in Figure S3, Supporting Information. The phonons were observed in the parallel polarizations (*z*(*xx*)$\bar{z}$ or *z*(*x*’*x*’)$\bar{z}$) but not in the cross polarizations (Figure S3, Supporting Information), which is consistent with the previous observation of longitudinal zone‐folded phonons in traditional compound semiconductors.^[^ ^12^ ^]^ Further, we could fit the doublets of the zone‐folded phonons using two Lorentzian oscillators, as shown in Figure S4, Supporting Information, to extract *ω*_ph_ as the average of the doublet frequencies. The experimentally observed *ω*_ph_ values were in good agreement with the theoretical expectation. We summarized the *ω*_ph_ values of the ZA phonons in previous studies on various heterostructures in **Table** **1**.^[^ ^7^, ^8^, ^15^, ^23^, ^28^, ^51^, ^52^, ^53^, ^54^, ^55^, ^56^ ^]^ Our result exclusively shows that the *ω*_ph_ of atomic‐scale oxide heterostructure can be largely modulated within the THz frequency range.

**Table 1:** Observed *ω*_ph_ of ZA phonons from previous experiments are compared to that in SRO/STO superlattice in the current study^[^ ^7^, ^8^, ^15^, ^23^, ^28^, ^51^, ^52^, ^53^, ^54^, ^55^, ^56^ ^]^

Material	*ω*_ph_ [THz]	Repetition number	Growth method	Measurement method	Reference
InGaN/GaN	0.66–1.23	14	Metal–organic chemical vapor deposition	Time‐resolved pump‐probe experiments	[51]
GaAs/AlAs	0.40–0.96	40	Molecular beam epitaxy	Time‐resolved pump‐probe experiments	[7]
GaAs/AlAs	0.50–0.61	60 or 80	Molecular beam epitaxy	Time‐resolved pump‐probe experiments	[52]
GaAs/AlAs	0.22–0.74	220	Metal–organic chemical vapor deposition	Time‐resolved pump‐probe experiments	[53]
GaAs/AlAs	0.15–0.90	11	Molecular beam epitaxy	Raman spectroscopy	[8]
InSe/hBN	0.02–0.15	1	mechanical exfoliation	Time‐resolved pump‐probe experiments	[54]
BaTiO_3_/SrTiO_3_	0.36–0.90	30 or 50	Pulse laser deposition	Raman spectroscopy	[15]
BaTiO_3_/SrTiO_3_	1.8	25	Molecular‐beam epitaxy	Raman spectroscopy	[28]
YBa_2_Cu_3_O_7−x_/La_1/3_Ca_2/3_MnO_3_	0.26–0.35	26	Laser molecular beam epitaxy	Time‐resolved pump‐probe experiments	[55]
YBa_2_Cu_3_O_7_/manganite compositions	0.15–0.54	10	Pulse laser deposition	Confocal Raman spectroscopy	[23]
SrTiO_3_/(SrRuO_3_ or SrIrO_3_)	0.50–0.97	5 or 10	Pulse laser deposition	Time‐resolved pump‐probe experiments	[56]
SrRuO_3_/SrTiO_3_	1.30–2.20	50	Atomic‐scale pulsed laser epitaxy	Confocal Raman spectroscopy	This work

The linear relation between *ω*_ph_ and reciprocal lattice spacing (*π*/*d*) further confirms the atomistic engineering of phonons in oxide superlattices, as shown in Figure 2f. The effective sound velocity, *υ*_s_, estimated from the linear relation between *ω*_ph_ and *π*/*d* can provide another experimental evidence. In superlattice configurations, the effective *υ*_s_ can be calculated by the weighted arithmetic average of the constituent layers within the supercell because the wavelength of the probing light is significantly larger than the u.c. length of the supercell. We obtained the effective *υ*_s_ of the SRO/STO superlattices to be approximately 6.7 nm ps^−1^ (6700 m s^−1^) at room temperature using a linear fit, as shown by the gray solid line in Figure 2f. We could not observe any meaningful temperature dependence of the effective *υ*_s_ (refer to blue empty circles in Figure 2f for *ω*_ph_ values at 10 K), this is also consistent with the temperature independence of *υ*_s_ of the SRO/STO heterostructure.^[^ ^40^ ^]^

**Table** **2** summarizes the reference *υ*_s_ values of SRO and STO, and the effective experimental *υ*_s_ values of the SRO/STO superlattices. From the density function calculations, it was predicted that *υ*_s_ of bulk STO and SRO are 8.0 and 6.6 nm ps^−1^,^[^ ^29^, ^30^ ^]^ respectively, which coincide with the values of 7.9 and 6.3 nm ps^−1^, respectively, obtained experimentally using optical spectroscopy.^[^ ^31^, ^32^ ^]^ We estimated the effective *υ*_s_ of the SRO/STO superlattices using the following equation, considering superlattice geometry,

(3) $$\begin{array}{c} \begin{array}{c} v_{s}=\frac{1}{4}\left(\frac{6}{8}+\frac{6}{10}+\frac{6}{12}+\frac{6}{14}\right)v_{s,\mathrm{SRO}}+\frac{1}{4}\left(\frac{2}{8}+\frac{4}{10}+\frac{6}{12}+\frac{8}{14}\right)v_{s,\mathrm{STO}} \end{array} \end{array}$$

**Table 2:** Sound velocities of SRO and STO from previous studies are compared to that in SRO/STO superlattice^[^ ^29^, ^30^, ^31^, ^32^ ^]^

Material		*d*_pc_ [nm]	*υ*_s_ [nm ps^−1^]	Method	Reference
Theory	SrTiO_3_	0.3940	8.0	Density functional theory	[29]
	SrRuO_3_	0.3914	6.6	Density functional theory	[30]
Experiment	SrTiO_3_	0.3905	7.9	Brillouin spectroscopy	[31]
	SrRuO_3_	0.3937	6.3	Ultrasonic pulse‐echo method	[32]
This work	SrRuO_3_/SrTiO_3_ superlattices	Systematically controlled	6.7	Confocal Raman spectroscopy	‐

Using the values of *υ*_s,SRO_ and *υ*_s,STO_ (*υ*_s_ of SRO and STO, respectively) from the previous theory and experiment (Table 2), the effective *υ*_s_ values of 7.1 and 6.9 nm ps^−1^ were deduced. These values are in excellent agreement with our experimentally obtained effective *υ*_s_ of 6.7 nm ps^−1^, as shown in Figure 2f. As *υ*_s_ represents the fundamental lattice properties of a crystal, defined by *υ*_s_ = $\sqrt{c/\rho }$ (where *c* is the elastic modulus of the material), our results also imply that the lattice properties of atomically thin SRO and STO layers within the superlattice are well preserved.

### 2.3 Emergent Polar Optical Phonons in the Superlattices

**Figure 3:**
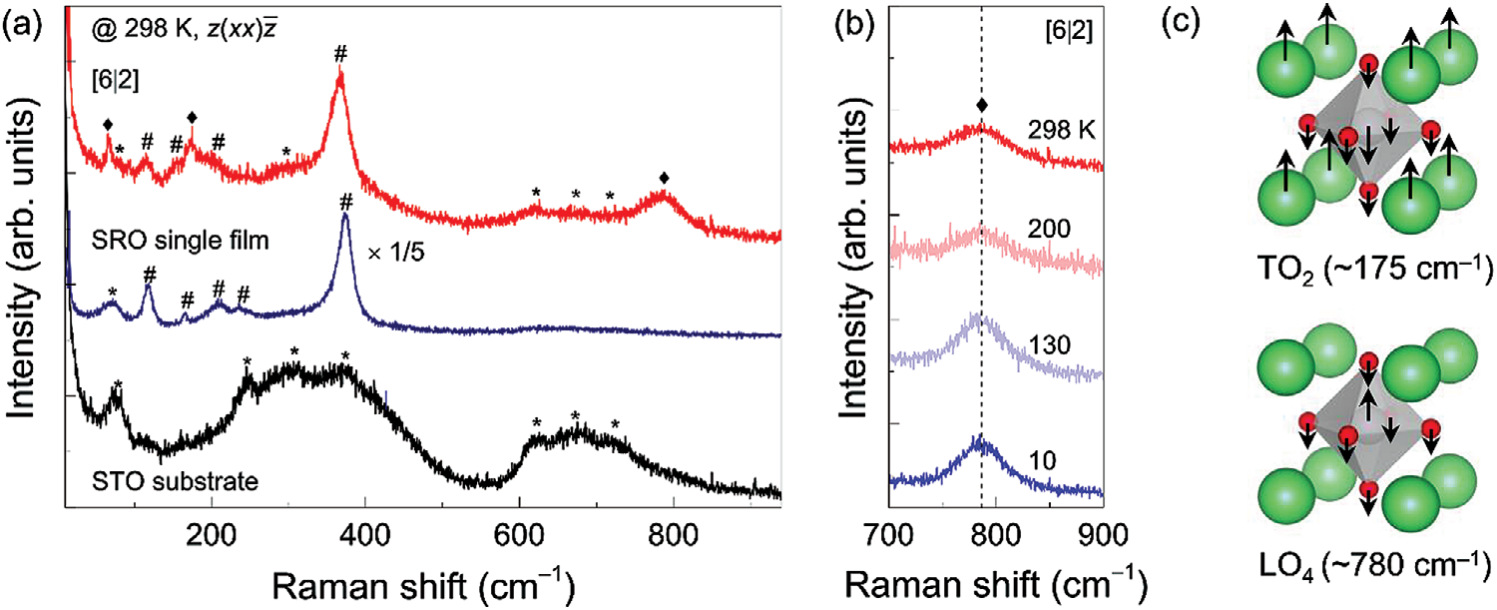
Observation of polar optical phonon mode in SRO/STO superlattices. a) Raman spectra for STO substrate, SRO single film, and superlattice. The spectra are measured with *z*(*xx*)$\bar{z}$ scattering configuration at room temperature. Raman intensity of SRO film is multiplied by 1/5. (We note that three phonon modes in the range of 600–750 cm^−1^ consistently exist in the Raman spectra of the SRO single film.) The asterisk (*), hash (#), and diamond (♦) symbols indicate the phonon assignments for STO substrate, SRO layers, and superlattices, respectively. b) Raman spectra for SRO/STO superlattice show temperature dependence of LO_4_ mode. c) The schematics show the polar phonon modes of TO_2_ and LO_4_ for TiO_6_ octahedra within STO. The arrows indicate the eigenvector of the phonon mode along [001].

Raman spectra in the higher frequency range exhibited an unexpected phonon mode for the SRO/STO superlattice (**Figure** **3**). Figure 3a compares the Raman spectrum of [6|2] superlattice with those of the SRO single film and STO substrate at room temperature. Seven Raman peaks of the STO substrate (indicated by asterisks) originate from the second‐order Raman scattering of STO.^[^ ^48^ ^]^ Five Raman peaks of the SRO single film (indicated by hashes) are the known phonon modes of the SRO layer.^[^ ^45^ ^]^ Most of the Raman peaks of the superlattice can be interpreted as optical phonon modes of the SRO and STO layers (asterisks and hashes) or ZA phonon modes (∼66 cm^−1^) as discussed earlier. However, the two Raman excitations at ∼175 and ∼780 cm^−1^ (diamonds) cannot be explained by the conventional Raman modes of individual SRO and STO. The surface plasmon‐polariton in the SRO/STO nanoribbons revealed a similar excitation at ∼800 cm^−1^.^[^ ^49^ ^]^ Yet, our measurement configuration does not allow the surface plasmon‐polariton at the SRO/STO interfaces because the polarization of the incident laser is orthogonal to that of the surface plasmon‐polariton. Furthermore, the temperature independence of the peak frequency, as shown in Figure 3b, does not support the surface plasmon‐polariton mode.

Figure 3c schematically shows the possible PO phonon modes emerging in the SRO/STO superlattice, which can explain the observed Raman peaks. Whereas the original cubic STO does not host any polar phonons, TO_2_ and LO_4_ PO phonons were observed when the inversion symmetry of the system breaks via various means including epitaxial strain, oxygen isotope doping, or external electric field.^[^ ^23^, ^50^, ^57^, ^58^, ^59^ ^]^ Indeed, the peak frequencies of the Raman modes of the superlattices coincide with the emergent PO phonons in the STO. Moreover, the Raman spectra of the superlattice at 10 K show significant enhancements of the peaks (Figure S5, Supporting Information), further supporting the appearance of the TO_2_ and LO_4_ phonons. For atomically thin superlattices, even when the ideal global inversion symmetry is preserved, local inversion symmetry can be intrinsically broken at the interfacial layers.^[^ ^26^, ^27^, ^47^ ^]^ From density functional theory calculations, it can also be inferred that the inversion symmetry at SRO/STO interfaces is intrinsically broken.^[^ ^60^ ^]^ Thus, we believe that the two Raman peaks at ∼175 and 780 cm^−1^ are TO_2_ and LO_4_ polar phonons of the atomically thin STO layer, originating from the local symmetry breaking of the superlattice structure.

## 3 Conclusions

In summary, we demonstrated atomistic engineering of phonons in deliberately designed oxide heterostructures. The atomically well‐defined periodicity of the oxide superlattices led to the backfolding of acoustic phonon dispersion in the presence of zone‐folded phonons in THz frequencies, which provides important implications for acoustic Bragg mirrors and cavities for generating coherent THz phonons. Furthermore, we systematically controlled the excitation energies over 2 THz via atomic‐scale precision thickness control. We also observed the Raman excitation of the polar optical phonon of STO at room temperature in the superlattices. The atomically designed superlattices intrinsically break the local inversion symmetry, and thus, the polar optical phonon modes of atomically thin STO layers can be stabilized and visualized. Our approach offers a facile method for the artificial engineering of phonons in functional oxides for future quantum phononics.

## 4 Experimental Section

### Atomic‐Scale Epitaxial Growth and Lattice Characterization

The SRO/STO superlattices were synthesized with six‐ and *y*‐u.c. of the SRO and STO layers, that is, [6|*y*] superlattice, at 750 °C in 100 mTorr of oxygen partial pressure using pulsed laser epitaxy. To enhance the Raman cross‐section of inelastic light scattering, the SRO/STO superlattices were used with 50 repetitions. Stoichiometric SRO and STO targets were ablated using a KrF laser (248 nm, IPEX868, Lightmachinery) with a laser fluence of 1.5 J cm^−2^ and a repetition rate of 5 Hz. X‐ray *θ*–2*θ*, off‐axis, and reciprocal space map measurements were performed using a high‐resolution PANalytical X'Pert X‐ray diffractometer. X‐ray rocking curve measurements show the excellent crystallinity of the superlattices even after 50 repetitions (Figure S2, Supporting Information).

### Raman Spectroscopy

The Raman spectra of SRO/STO superlattices were obtained using a confocal micro‐Raman (Horiba LabRam HR800) spectrometer with 632.8 nm (1.96 eV) HeNe laser. Temperature‐dependent measurements were performed under vacuum using a custom‐built optical cryostat. A grating with 1800 grooves per mm and a focused beam spot with a size of 5 µm were used. The power of the laser beam was kept below 0.3 mW to avoid any laser heating effects. The *z*‐directional beam position was deliberately controlled to achieve optimal focus on the superlattice samples for measuring high‐quality Raman spectra using a backscattering geometry.^[^ ^61^ ^]^ Conventional Raman spectroscopy for wide‐bandgap insulating oxide thin‐films often required the resonant excitation of laser. However, our confocal spectroscopy allowed to probe clear Raman scattering intensities from atomically thin oxide layers.^[^ ^19^, ^48^ ^]^ The coherent supercell structures, up to 50 repetitions (thicknesses of 160–280 nm), also increased the inelastic light scattering cross‐section, which was sufficient for detecting the Raman spectra of the ZA phonon modes.

## Conflict of Interest

The authors declare no conflict of interest.

## Supporting information

Supporting Information

## Data Availability

Research data are not shared.
