# Supplementary material for: Atomistic Engineering of Phonons in Functional Oxide Heterostructures
Source: Adv Sci (Weinh). 2022 Jan 17;9(7):2103403. doi: 10.1002/advs.202103403 (PMC8895146; doi:10.1002/advs.202103403)
Supplement: Supplementary file 1 — Supporting Information [file ADVS-9-2103403-s001.pdf]

WILEY-VCH

ADVANCED SCIENCE
Open Access

Supporting Information

for *Adv. Sci.*, DOI: 10.1002/advs.202103403

Atomistic Engineering of Phonons in Functional Oxide Heterostructures

*Seung Gyo Jeong, Ambrose Seo, and Woo Seok Choi**

Supporting Information

Atomistic Engineering of Phonons in Functional Oxide Heterostructures

Seung Gyo Jeong, Ambrose Seo, and Woo Seok Choi*

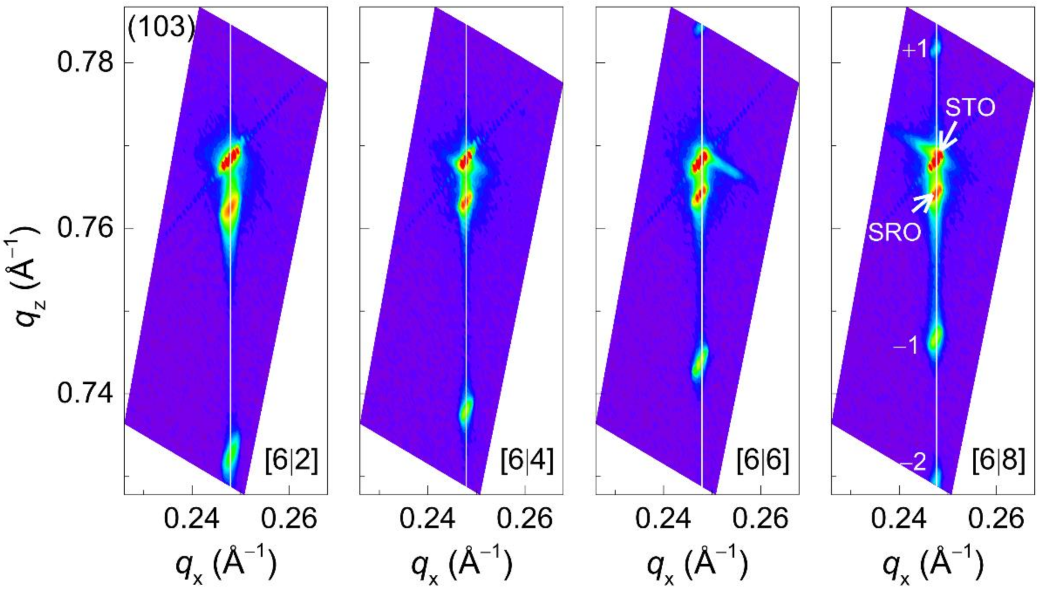

Figure S1. XRD RSMs of the SRO/STO superlattices. RSMs of the [6|*y*] superlattices with different *y* were measured around the STO (103) Bragg reflection. The Bragg peaks of the superlattices (±*n*) correspond to atomically-well defined periodicities of superlattices. The vertical lines indicate a fully strained state of superlattices.

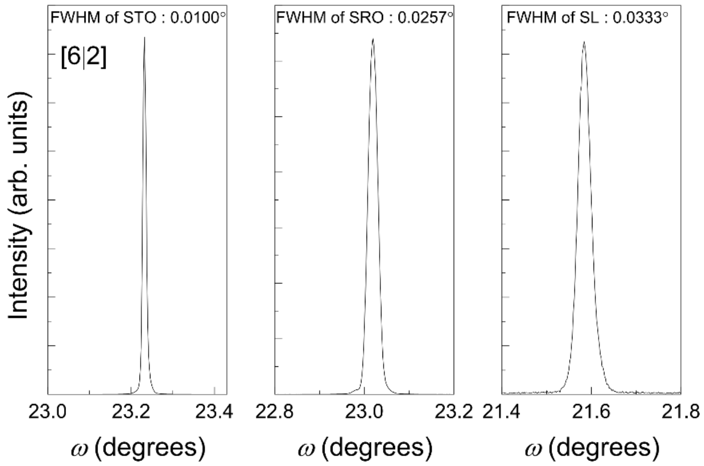

Figure S2. XRD rocking curve measurements of the SRO/STO superlattice. Rocking curve measurements of [6|8] superlattice were recorded around (002) Bragg reflection of each layer. The full-width-at-half-maximum (FWHM) value of SRO and superlattice peaks are comparable to that of the STO substrate. This result shows the excellent crystallinity of our oxide superlattices.

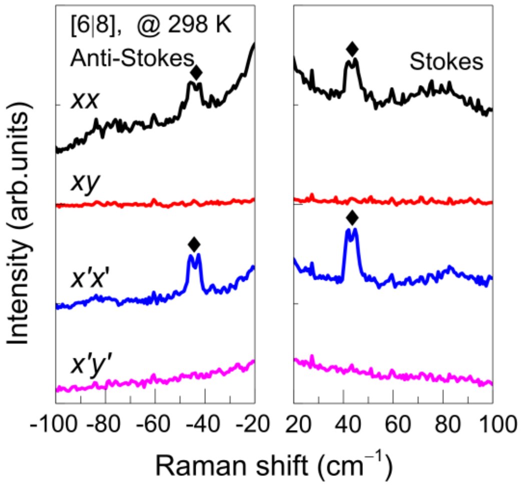

Figure S3. Polarization-dependent Raman spectra of the SRO/STO superlattices. Polarized Raman spectra of [6|8] superlattices were measured at room temperature. Diamonds (♦) indicate the zone-folded acoustic phonons. Both Stoke and anti-Stoke scattering assure the existence of zone-folded acoustic phonon mode in the superlattice. Polarization dependence of the phonon is consistent with previous longitudinal zone-folded phonon.[12]

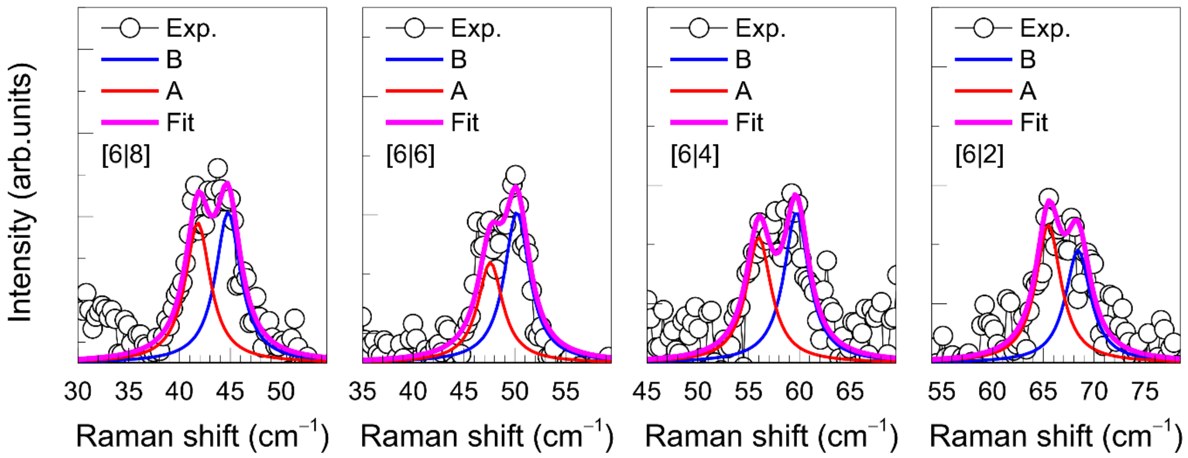

Figure S4. Lorentzian fitting for zone-folded acoustic phonon modes. The *y*-dependent doublet of folded acoustic phonon ω_{SL}s of [6|*y*] superlattices were obtained by Lorentzian fitting using two peaks.

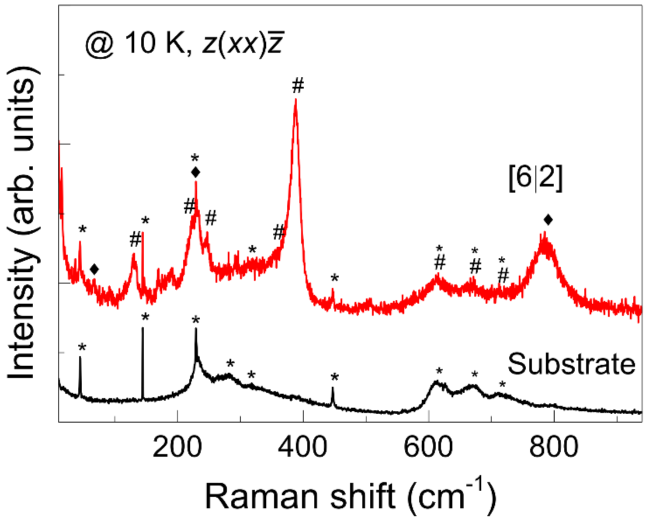

Figure S5. Raman spectra of the SRO/STO superlattice and STO substrate, measured at 10 K. The asterisk (*), hash (#), and diamond (♦) symbols indicate the phonon assignments for STO, SRO layers, and superlattices, respectively.
